# Is the Sense of Taste Impaired by Plates?

**Published:** 1868-05

**Authors:** J. P. H. Brown

**Affiliations:** Augusta, Ga.


### IS THE SENSE OF TASTE IMPAIRED BY PLATES?

BY DR. J. P. H. BROWN.

THIS question is very often put to the Dentist, and often answered very indefinitely. In order to answer a question clearly and satisfactorily, it is necessary for us to have a clear knowledge of the subject matter involved in the question.

Although the number and distribution of the nerves of taste have been a subject of great controversy, yet I believe it is now unanimously agreed that the *glosso-pharyngeal* of the eighth pair of nerves, and the *lingual branch* of the inferior maxillary nerve of the fifth pair, are the nerves of this special function. The first, supplying taste to the posterior third of the tongue and *soft* palate; and the second, to the anterior two-thirds of this organ. The mental impressions leading to the perception of taste result, no doubt, from certain changes in the internal condition of the nerves produced by the contact of sapid substances with the papillæ of the tongue, in which the extremities of these nerves are distributed. The nature and philosophy of these changes is not well understood. Some physiologists, Raspail among the number, explain it by the theory of galvanic action. The tongue they regard the *true organ of taste*, and the "positive pole;" the hard palate, lips, and teeth, the "negative;" and the sapid substance, which must be in solution in the secretions of the organ of taste, completes the circle. They use the simple experiment of placing the tip of the tongue between two metals, such as zinc and silver, to illustrate their theory. Every time the edges of the metals are brought together, a strong alkaline taste is perceived.

Without fully subscribing to the above theory, I think it is very certain that a third body (a "negative pole") is necessary in order to enable the tongue to exhibit properly this function. If the end of the tongue be immersed in a sapid solution, and not brought in contact with the hard palate, or with the teeth and lips, there will be a very imperfect manifestation of taste. But as soon as the tongue is brought to bear against any of the parts that surround it, the taste becomes developed. It may be well here to remark that in conducting this experiment the sapid substance should be devoid of odor; for much of the perfection of the sense of taste depends upon the simultaneous action of the sense of smell. This is shown by the simple experiment of protruding the tongue and touching its tip with a little tannic acid. The taste of the acid is immediately recognized. Now close the nostrils and the taste is gone. Remove the fingers from the nose and the taste returns.

The *rugæ* of the hard palate being endowed with only nerves of sensation and not with those of taste, can assist the positive organ of taste—the tongue—only by diffusing the sapid matter over its sensitive surface by compression, friction, and motion, in order to bring the substance into contact with the delicate papillæ. If a piece of thick writing paper be slightly moistened, and on one side a little sugar be placed, and on the other a few grains of oxalic acid, and then carefully inserted into the mouth with the acid side up against the hard palate, and the tongue brought in contact with the under side, the sweet taste of the sugar will at once be recognized by the tongue, but no taste of the acid will be perceived by the rugæ. Now remove it, and thoroughly rinse the mouth, and reverse the paper with the sugared side up, the tongue will instantly detect the acid; but the hard palate will be unconscious of the presence of the sugar. This experiment will be more successful by using in place of the paper a very thin pliable piece of pure gold ribbon, such as goldbeaters get out for making gold foil.

From these experiments and from the distribution of the nerves of taste, we can safely conclude that the rugæ of the hard palate assists the tongue in the function of taste only in a negative manner. When a plate is first placed in the mouth the part of the palatine arch covered by it no longer feels the impression of the tongue, and the tongue no longer feels the impression of the rugæ. This creates at first a confusion of feeling which is often confounded with a loss of taste. But as the rugæ are only negative agents so also is the plate. It assists the tongue by friction and pressure to detect sapid matters in precisely the same manner as the former. Hence if the plate be composed of a material that is not capable of being acted on by the oral secretions; that will not absorb; is perfectly inodorous; and is so constructed that the wearer can keep it perfectly clean, it does not in any way impair the taste. From my own observation, and this has been somewhat extensive, I venture the assertion that in ninety-nine cases out of every hundred where there is any complaint of loss of taste by wearing a plate, that the material of which it is constructed is either chemically affected by the secretions, or absorbs foul matters into its substance, or has interstices and places for their lodgment beyond the reach of removal. Rubber work (offensively odorous) after being worn a short time absorbs and retains so many different matters and odors that the nerves of taste become blunted and confounded by the heterogeneous mass of filth, so that it is impossible for them to distinguish correctly different tastes. On this impairment of taste by commingling sapid matters, Mr. Kirkes in his *Manual of Physiology* very correctly observes: "Very distinct sensations of taste are frequently left after the substances which excited them have ceased to act on the nerve; and such sensations often endure for a long time, and modify the taste of other substances applied to the tongue afterward. Thus, the taste of sweet substances spoils the flavor of wine, the taste of cheese improves it. There appears, therefore, to exist the same relation between tastes as between colors, of which those that are opposed or complimentary render each other more vivid, though no general principles governing this relation have been discovered in the case of tastes. * * * Frequent and continued repetition of the same taste renders the perception of it less and less distinct, in the same way that a color becomes more and more dull and indistinct, the longer the eye is fixed upon it. Thus, after frequently tasting first one and then the other of two kinds of wine, it becomes impossible to discriminate between them."

Dentists can not be too careful to impress the minds of their patients with the importance of keeping plates *clean*. Rubber plates are more difficult to keep clean than any other. The mucus and foreign matters seem to have a peculiar attraction for it, and adhere so tightly that soap and a brush are not always sufficient to remove them. In such cases it is not uncommon to hear complaints of loss of taste.

AUGUSTA, Ga.

